# Effect of ceftazidime-avibactam combined with different antimicrobials against carbapenem-resistant *Klebsiella pneumoniae*

**DOI:** 10.1128/spectrum.00107-24

**Published:** 2024-05-07

**Authors:** Yun Wu, Wei Yu, Xiaobing Chu, Jingjia Zhang, Peiyao Jia, XiaoYu Liu, Ying Zhu, YingChun Xu, Qiwen Yang

**Affiliations:** 1 Department of Clinical Laboratory, State Key Laboratory of Complex Severe and Rare Diseases, Peking Union Medical College Hospital, Beijing, China; 2 Graduate School, Peking Union Medical College, Chinese Academy of Medical Sciences, Beijing, China; University of Debrecen, Debrecen, Hungary

**Keywords:** antimicrobial agent combinations, ceftazidime-avibactam, checkerboard test, time-kill curve assay, carbapenem-resistant* Klebsiella pneumoniae*

## Abstract

This study aimed to assess the *in vitro* efficacy of ceftazidime-avibactam (CZA) in combination with various antimicrobial agents against carbapenem-resistant *Klebsiella pneumoniae* (CRKP). We selected 59 clinical CRKP isolates containing distinct drug resistance mechanisms. The minimum inhibitory concentrations (MICs) of meropenem (MEM), colistin (COL), eravacycline (ERA), amikacin (AK), fosfomycin (FOS), and aztreonam (ATM), both individually and in combination with CZA, were tested using the checkerboard method. The interactions of antimicrobial agent combinations were assessed by fractional inhibitory concentration index (FICI) and susceptible breakpoint index (SBPI). The time-kill curve assay was employed to dynamically evaluate the effects of these drugs alone and in combination format. In the checkerboard assay, the combination of CZA+MEM showed the highest level of synergistic effect against both KPC-producing and carbapenemase-non-producing isolates, with synergy rates of 91.3% and 100%, respectively. Following closely was the combination of FOS+CZA . For metallo-beta-lactamases (MBLs) producing strains, ATM+CZA displayed complete synergy, while the combination of MEM+CZA showed a synergy rate of only 57.14% for NDM-producing strains and 91.67% for IMP-producing strains. In the time-kill assay, MEM+CZA also demonstrated significant synergistic effects against the two KPC-2-producing isolates (Y070 and L70), the two carbapenemase-non-producing isolates (Y083 and L093), and the NDM-1-producing strain L13, with reductions in log_10_ CFU/mL exceeding 10 compared to the control. Against the IMP-producing strain Y047, ATM+CZA exhibited the highest synergistic effect, resulting in a log_10_ CFU/mL reduction of 10.43 compared to the control. The combination of CZA and MEM exhibited good synergistic effects against KPC-producing and non-enzyme-producing strains, followed by the FOS+CZA combination. Among MBL-producing strains, ATM+CZA demonstrated the most pronounced synergistic effect. However, the combinations of CZA with ERA, AK, and COL show irrelevant effects against the tested clinical isolates.

**IMPORTANCE:**

Our study confirmed the efficacy of the combination CZA+MEM against KPC-producing and non-carbapenemase-producing strains. For metalloenzyme-producing strains, CZA+ATM demonstrated the most significant synergy. Additionally, CZA exhibited a notable synergy effect when combined with FOS. These combination therapies present promising new options for the treatment of CRKP infection.

## INTRODUCTION

Carbapenem-resistant Enterobacterales (CRE) poses an urgent global public health threat, with more than 1,100 documented deaths, as reported in a 2019 antibiotic resistance publication by the U.S. Centers for Disease Control and Prevention (CDC) (1). Notably, CRE, including carbapenem-resistant *Klebsiella pneumoniae* (CRKP), is associated with higher mortality compared with infections caused by carbapenem-susceptible *Enterobacterales* infections (2). The primary resistance mechanisms of CRKP are the production of carbapenemases, including class A enzymes (e.g., KPC), class B enzymes (e.g., NDM and IMP), or class D enzymes (e.g., OXA-48). Additionally, the loss of porins, specifically ompK 35 and/or ompK36, represents another pivotal resistance mechanism in CRKP (3). In China, among the prevalent CRKP strains, the most common are KPC-procedures strains (4), followed by NDM-procedures strains (4), while strain-producing VIM and OXA (5) enzymes are relatively rare.

Ceftazidime-avibactam (CZA) is a novel combination of a third-generation cephalosporin and a novel β-lactamase inhibitor (6), demonstrating efficacy against CRKP. Although CZA is currently a significant drug for the treatment of CRKP, several researches have elucidated the emergence of resistance to CZA (7). Some investigations showed the emergence of CZA resistance due to a single alternative mutation in the KPC-2 or KPC-3 gene (8, 9). With the increasing use of this drug, the prevalence of resistance is expected to escalate, rendering monotherapy less effective for severe infections. Combination therapy has demonstrated effectiveness against a broad spectrum of resistant organisms, ensuring maximum antimicrobial efficacy (9). This approach not only diminishes the reliance on single agents but also mitigate the emergence of drug resistance. Notably, synergistic effects between different antibacterial drugs have been observed, further emphasizing the potential benefits of combination strategies.

This study aimed to investigate the efficacy of CZA, both as a standalone treatment and in combination with other clinically recommended drugs, including meropenem (MEM), colistin (COL), eravacycline (ERA), amikacin (AK), fosfomycin (FOS), and aztreonam (ATM). Notably, the combination of ATM and CZA was exclusively assessed in strains producing MBL.

## MATERIALS AND METHODS

### Bacterial strains and culture media

A total of 59 strains were selected from 11 teaching hospitals across China during the period spanning 2015 to 2018. These clinical isolates *K. pneumoniae* exhibited diverse resistance mechanisms, including KPC producers (22/59), IMP producers (12/59), NDM producers (14/59), and non-carbapenemase procedures but had porin ompK35 and/or ompK36 loss (11/59).

All strains were identified using the MALDI-TOF MS apparatus (Bruker Biotyper; Bruker Daltonik, Bremen, Germany). Mueller-Hinton broth and Luria-Bertani agar broth were used for susceptibility testing and time-kill experiments, respectively.

### MLST and carbapenemase detection

Multilocus sequence typing (10) (MLST) was performed as described on the Pasteur Institute MLST website (https://bigsdb.pasteur.fr/), including DNA sequencing analysis of the seven housekeeping genes. The carbapenemase gene (11) (multiplex) probe-based Hi-Media Hi-PCR kit was used to detect specific regions of the gene encoding the carbapenemase enzymes (Table S1). The phenotypic Carbapenem Inactivation Method (CIM) was conducted following the CLSI recommended guidelines to examine whether strains could produce carbapenemase. Briefly, 1 µL of overnight-cultured bacteria was mixed with 2 mL of tryptic soy broth (TSB), and 10 mg of meropenem disk was added; incubated the suspension at 37°C for 4 h. After incubation, the disk was removed from the suspension using an inoculation loop, placed on a Mueller-Hinton agar plate inoculated with a susceptible *Escherichia coli* indicator strain (ATCC 29522) and subsequently incubated at 35°C. Disks incubated in suspensions that do not contain carbapenemases would yield a clear inhibition zone (12).

### Antimicrobial susceptibility test

Minimum inhibitory concentrations (MICs) of ERA, FOS, MEM, COL, AK, ATM, and CZA were determined by the broth microdilution method according to the CLSI recommendations. The concentration ranges for each drug are as follows: MEM (0.5–64 µg/mL), COL (0.125–16 µg/mL), AK (0.25–32 µg/mL), FOS (2–256 µg/mL), ERA (0.064–8 µg/mL), ATM (0.25–32 µg/mL), and CZA (0.031/4–4/4 µg/mL).

The results were interpreted according to CLSI criteria. *E. coli* strain ATCC 25922 and *Pseudomonas aeruginosa* ATCC 27853 were used as quality control organisms. The MIC results were measured after 16–20 h of incubation in the air at 35℃ and interpreted as susceptible, intermediate, or resistant according to CLSI guidelines.

### Checkerboard test

We used CZA as the basic antibacterial agent in combination with other drugs. Six combinations were tested including CZA+MEM, CZA+AK, CZA+COL, CZA+ERA, CZA+ATM and CZA+FOS. Taking the CZA+MEM combination as an example to demonstrate the preparation of the drug susceptibility test plates, the lateral direction represents the MEM gradient dilution direction, while the longitudinal direction represents the CZA gradient dilution direction. The prepared starting solution is subjected to a series of two-fold dilution, and 25 µL of the solution was added to each well, forming drug susceptibility test plates with the concentration range of CZA from 0.031 μg/mL to 4 μg/mL and MEM from 0.5 μg/mL to 64 μg/mL. These plates were then stored at −80℃ for standby. The test strains were inoculated into the drug susceptibility test plates at a final concentration of 5 × 10^5^ CFU/mL, then incubated the plates for 20 hours at 35℃ to judge MIC.

The efficacy of the antimicrobial combination was evaluated using the fractional inhibitory concentration index (FICI). The FICI was calculated using the following formula: FICI = (MIC of agent A in combination/MIC of agent A alone) + (MIC of agent B in combination/MIC of agent B alone) (13). The results were interpreted as follows: FICI ≤0.5 indicated synergistic, 0.5＜ FICI ≤4 implied irrelevant, FICI >4 is antagonistic (14).

Additionally, we employed the susceptible breakpoint index (SPBI) to evaluate the interaction of antimicrobial agents. SPBI was calculated as follows: SBPI＝ (susceptible breakpoint A/MIC of A in combination) + (susceptible breakpoint B/MIC of B in combination) (15).

### Time–kill assay

In order to dynamically assess the bactericidal activity of the antimicrobials, we chose six representative strains to conduct time-kill assay, which showed the synergistic effect for most of the six drug combinations. Detailed information about these six strains is presented in Table 1. The drug concentrations were determined based on reported blood concentrations that could be achieved for each drug. The individual drug concentrations, both in isolation and in combination, are as follows: MEM 8 µg/mL, COL 0.25 µg/mL, ERA 0.25 µg/mL, AK 16 µg/mL, FOS 32 µg/mL, ATM 1 µg/mL, CZA 0.5/4 µg/mL.

**TABLE 1:** The characteristics of isolates for time-killing test^*a*^

Strain no.	Carbapenemase	MIC [Interpretation]						
		CZA	AK	COL	ERA	FOS	MEM	ATM
L70	KPC-2	0.031[S]	128[R]	0.25[S]	0.5[S]	8[S]	8[R]	–^*b*^
Y070	KPC-2	2[S]	128[R]	0.25[S]	0.25[S]	1024[R]	128[R]	–
Y083	Negative	4[S]	128[R]	0.25[S]	0.25[S]	128[R]	16[R]	–
L093	Negative	4[S]	0.5[S]	0.25[S]	4[R]	256[R]	32[R]	–
L013	NDM-1	64[R]	8[S]	0.25[S]	0.5[S]	512[R]	64[R]	64[R]
Y047	IMP-8	64[R]	128[R]	2[R]	1[S]	512[R]	4[R]	64[R]

^*a*^ MEM, meropenem; ERA, eravacycline; FOS, fosfomycin; COL, colistin; AK, amikacin; ATM, aztreonam; CZA, ceftazidime-avibactam;R,resistant; S,Sensitive.

^*b*^ "–" indicates that the drug tested in KPC-producing as well as non-carbapenemase-producing strains did not include the AZA.

Firstly, prepare 0.5 McFarland Standard Turbidity of colonies growing in the logarithmic growth overnight, and dilute with MHBII by 200 folds to 5 × 10^5^ CFU/mL bacterial solution. Secondly, prepare the mixture of bacteria and drug with concentration as listed above and incubate the mixture in a shaker at 35℃. At 0, 2, 4, 8, and 24 h, obtain aliquots of 0.1 mL from each tube, serially dilute them in 0.9% sodium chloride, and inoculate 10 µL on LB agar plates. After 24 h of incubation at 37°C, the plates were taken out, and the colonies were calculated (16). Time-kill curves were created by plotting mean colony counts (log_10_ CFU/ml) versus time to compare the 24-h killing effects of monotherapy and combination antimicrobial exposure.

The lower limit of detection is 2.0 log_10_ CFU/ mL. Bacterial concentrations less than 2.0 log_10_ CFU/mL are considered as 2.0 log_10_ CFU/mL. If the 24-h concentration is decreased by 2.0 log_10_CFU/mL compared with the most active antibiotic alone, it indicates synergy. Irrelevance is defined as a <2 log_10_ CFU/mL increase or decrease at 24 h for the drug combination in comparison with the most active antibiotic alone. Antagonism is defined as a＞2log_10_CFU/mL increase at 24 h for the drug combination when compared with the drug alone (17).

## RESULTS

### Checkerboard test

The results of the checkerboard combination test are presented in Table 2, and detailed FICI results are listed in Table S2.

**TABLE 2:** The results of checkerboard combination test^*a*^

		AK + CZA	COL + CZA	ERA + CZA	FOS + CZA	MEM + CZA	ATM + CZA
KPC(*n* = 22)	Synergy *N*(%)	4(18.18%)	0(0.00%)	3(13.64%）	10(43.37%)	20(91.30%)	——^*b*^
	Irrelevant *N*(%)	18(81.82%)	22(100.0%）	19(86.36%）	12(56.52%）	2(8.70%）	——
	Antagonism *N*(%）	0(0.00%)	0(0.00%)	0(0.00%)	0(0.00%)	0(0.00%)	——
carbapenemase-non-producing isolates (*n* = 11)	Synergy *N*(%)	0(0.00%)	0(0.00%)	2(18.00%）	3(27.00%)	11(100.0%）	——
	Irrelevant *N*(%)	11(100.0%）	11(100.0%）	9(82.00%）	8(73.00%）	0(0.00%)	——
	Antagonism *N*(%）	0(0.00%)	0(0.00%)	0(0.00%)	0(0.00%)	0(0.00%)	——
NDM(*n* = 14)	Synergy *N*(%)	3(21.40%）	0(0.00%)	0(0.00%)	9(64.28%）	8(57.14%）	14(100.00%)
	Irrelevant *N*(%)	11(78.57%)	14(100.00%)	14(100.00%)	5(35.72%)	6(42.86%)	0(0.00%)
	Antagonism N(%）	0(0.00%)	0(0.00%)	0(0.00%)	0(0.00%)	0(0.00%)	0(0.00%)
IMP (*n* = 12)	Synergy *N*(%)	7(58.30%)	2(16.70%)	4(33.33%)	9(75.00%)	11(91.67%)	12(100.00%)
	Irrelevant *N*(%)	5(42.70%)	10(83.30%)	8(66.67%)	3(25.00%)	1(8.33%)	0(0.00%)
	Antagonism *N*(%）	0(0.00%)	0(0.00%)	0(0.00%)	0(0.00%)	0(0.00%)	0(0.00%)

^*a*^ MEM, meropenem; ERA, eravacycline; FOS, fosfomycin; COL, colistin; AK, amikacin; ATM, aztreonam; CZA, ceftazidime-avibactam.

^*b*^ "——” indicates that the drug tested in KPC-producing as well as non-carbapenemase-producing strains did not include the AZA.

Against KPC carbapenemase-producing isolates, CZA+MEM combination showed the most effective interaction with a synergy rate of 91.30%, followed by CZA+FOS at 43.37%, and CZA+ERA at 13.64%. In contrast, CZA+AK and CZA+COL were mostly irrelevant. Notably, CZA+MEM exhibited synergy in 100.00% (11/11) of carbapenemase-non-producing isolates, while CZA+FOS showed a 27.00% synergistic activity, and CZA+ERA exhibited 18.00% synergy.

Against MBL-producing strains, ATM+CZA displayed remarkable synergy with a rate of 100.00%. Against IMP-producing strains, MEM+CZA a synergy rate of 91.67%, ranking second only to ATM+CZA. Following closely were FOS+CZA and AK+CZA, which displayed synergy rates of 75.00% and 58.30%, respectively. In the case of NDM-producing strains, FOS+CZA and MEM+CZA showed synergy rates of 64.28% and 57.14%, respectively.

In conclusion, the results indicated that MEM+CZA exhibited a strong synergistic effect in all strains, except for NDM-producing strains, where the synergistic rate was only 57.5%. In contrast, the synergistic rate exceeded 90.0% in other strains. Notably, against all MBL-producing strains, which were resistant to CZA and ATM when used individually, the combination of CZA+ATM displayed synergistic effects. Among these combinations, CZA+FOS displayed higher antibacterial activity against MBL-producing strains, with a synergy effect of over 60%, while for KPC producers and non-carbapenemase procedures, the range of synergy rates was 20% to 43%. CZA+AK, CZA+COL and CZA+ERA primarily showed irrelevant effect, with synergy rates ranging from 0.0% to 58.3%. None of the combinations showed antagonism.

The data of SBPI are presented in Table 3. Regarding the combination of CZA+MEM, the median of SBPI is 504, and the mean is 503.75, both of which are higher than other drug combinations among the KPC producers. However, in MBL-producing strains, the SBPI of CZA+MEM ranged from 0.265 to 75, which is lower than the combination of CZA+ATM. This suggests that CZA+ATM may exert a stronger synergistic effect for MBL-producing strains. There is no significant difference in SBPI among the other combinations.

**TABLE 3:** Susceptible breakpoint index (SBPI) of antimicrobial combinations tested against *Klebsiella pneumoniae* isolates with different resistance mechanisms^*a*^

		AK + CZA	COL + CZA	ERA + CZA	FOS + CZA	MEM + CZA	ATM + CZA
KPC (*n* = 22)	Range	32–128	16–397	20–564	4–565	502–504	——^*b*^
	Mean	72	48	101	66	503.7	——
	Median	75	94	101	33	504	——
carbapenemase-non-producing isolates (*n* = 11)	Range	12–96	17–80	8–68	9–80	502–504	——
	Mean	68	41	39	32	504	——
	Median	60	43	41	39	503.6	——
NDM (*n* = 14)	Range	0.5–80	8.5–72	40.25–72	0.375–68	0.265–75	64.5–80
	Mean	59.53	60.65	61.44	52.51	11.87	73.58
	Median	72	72	67	68	4.375	72
IMP (*n* = 12)	Range	0.5–128	16.5–72	16.5–72	1.5–68	1–64.25	72–80
	Mean	53.42	53.37	47.77	45.87	13.54	74
	Median	57	68	64.375	64.375	5	80
						

^*a*^ MEM, meropenem; ERA, eravacycline; FOS, fosfomycin; COL, colistin; AK, amikacin; ATM, aztreonam; CZA, ceftazidime-avibactam.

^*b*^ "——” indicates that the drug tested in KPC-producing as well as non-carbapenemase-producing strains did not include the AZA.

### Time–kill assay

**Fig 1:**
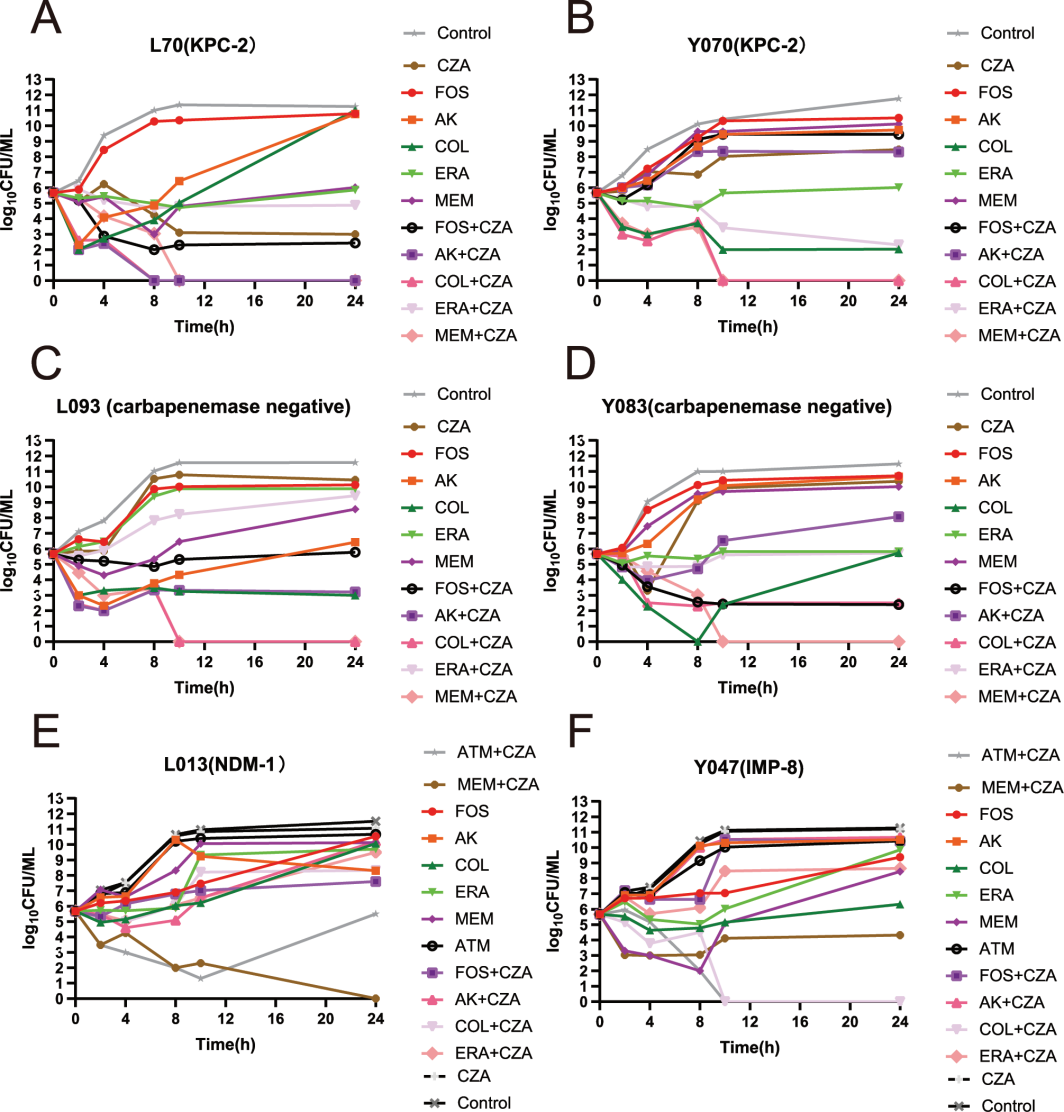
*In vitro* time–kill assay using ceftazidime-avibactam (CZA) alone or combine with meropenem (MEM), colistin (COL), eravacycline (ERA), amikacin (AK), aztreonam (ATM), and fosfomycin (FOS) against CRKPs with different resistance mechanisms. *The combination of ATM+CZA only tested in MBL-producing strains (L013 and Y047). * The drug concentrations are listed as follow: MEM 8 μg/ml, COL 0.25 μg/ml, ERA475 0.25 μg/ml, AK 16 μg/ml, FOS 32 μg/ml, ATM 1μg/ml, CZA 0.5/4 μg/ml. Carbapenem-resistant *Klebsiella pneumoniae,* CRKP*.*

The 24 h time-kill results are displayed in Fig. 1.

Against KPC-2-producing isolate L70, antimicrobial combination CZA+AK, CZA+MEM, and CZA+COL, showed a highly synergistic effect. Compared with the most effective single drug showed a decrease of 3 log_10_ CFU/ml. Against KPC-2-producing isolate Y070, the combinations of CZA+FOS, CZA+ERA and CZA+MEM showed a stronger synergistic effect, with log_10_ CFU/mL decreases of 6.45, 3.29, and 8.47, respectively, compared to the most active component of each single drug.

Against carbapenemase-non-producing isolate Y083, the combinations of CZA+AK, CZA+COL, CZA+FOS, and CZA+MEM both showed synergistic effect, and, among them, CZA+MEM was the most effective with log_10_ CFU/mL decreases of 11.49. Against carbapenemase-non-producing isolate L093, the combinations of CZA+COL, CZA+FOS, and CZA+MEM both showed synergistic effect, and, among them, CZA+MEM was the most effective with log_10_ CFU/mL decreases of 11.58.

Against MBL-producing strains Y074 and L013, the combinations of CZA+COL, CZA+MEM, and CZA+ATM both showed synergy effect. For the Y047 strain, which produces IMP enzyme, CZA+ATM was the most effective with log_10_CFU/mL decreases of 10.43 compared with the control. And for the NDM enzyme producer isolate L13, CZA+MEM showed the greatest effect with log_10_ CFU/mL decreases of 10.22.

In summary, CZA+MEM showed highly synergistic effect on all seven isolates and ATM+CZA displayed synergy effect in MBL-producing strains. Both of them are consistent with the results of the checkerboard test.

## DISCUSSION

The global prevalence of CRKP has gained significant attention in recent years, with numerous studies shedding light on its occurrence across different regions. For example, in a study of Sreeja K (11) CRKP incidence rates in various parts of India ranging from 14% to 69%. Similarly, Yunqing Qiu’s study found a prevalence of carbapenem resistance in pediatric bloodstream infection is 12.79% (18). Another study (19) indicated that about 50% of *K. pneumoniae* are producers of extended-spectrum beta-lactamase (ESBL). Furthermore, compared to non-ESBL-producing *K. pneumoniae*, patients infected with these pathogens experience increased mortality, primarily due to a delay in the administration of effective therapy (20, 21). Therefore, the rational choice of antimicrobial drugs is crucial.

CZA has been used as a frontline treatment for CRKP infections. However, it is crucial to note the increasing resistance rate to CZA. In a previous study, the overall resistance rate of *Enterobacterales* to CZA was <0.6%; however the resistance rate increased to 16.7%–21.0% for CRKP (22). Several studies have reported that the resistant rate of CZA against *K. pneumoniae* was 3.7% (32/872) (23) and 2.5% (24), which are higher than it first been used. Unfortunately, there are few clinical studies on the effects of CZA in combination with other drugs; although some previous studies had reported that about 3.5% cases of CRKP culture positivity persisted when using CZA alone, but the infections were managed with drug combinations (25). Our study revealed that CZA+MEM and CZA+ATM exhibited significant synergistic effects against KPC-2-producing and MBL-producing strains, respectively.

Avibactam is a class A and partial class D carbapenemase inhibitor which re-establishes susceptibility to carbapenems (26). MEM, a carbapenem antibiotic, binds to penicillin-binding proteins (27), thereby interfering the building of bacterial cell wall and exerting an antibacterial effect. So it’s logical that CZA+MEM against KPC-producing isolates showed 91.30% synergy effect (28). However, further researches are warranted to elucidate the underlying mechanisms contributing to the observed 100% synergy effect of CZA+MEM in non-enzyme producing but OmpK35 and OmpK36 porin-deficient isolates. In this study, MEM+CZA also displayed certain synergistic efficiency against MBL-producing strains, especially for IMP-producer, with a synergistic rate of 91.67%. This may be attributed to the fact that the 12 IMP-producing strains showed a lower resistance level to MEM compared to the NDM-producing strains, with 75% of the strains having a MIC of less than 16 µg/mL, but all of the NDM producer had a MIC of 64 µg/mL against MEM. In MBL-producing strains, the synergistic effect between MEM and CZA can be attributed to MEM serving as a substrate for the metalloenzyme, leading to the consumption of the enzyme and a subsequent reduction in its concentration. The provided CZA can inhibit the remaining metal enzymes to achieve a synergistic effect.

ATM is the only beta-lactamases stable to class B metalloenzymes, but it can be hydrolyzed by both class A and C beta -lactamases. In 2014, some researchers (29) had proposed a treatment option of avibactam plus ATM. They hypothesized that avibactam would be able to inhibit the co-existing ESBL and AmpC enzymes; thus, ATM could maintain its antibacterial activity. The study confirmed that CZA+ATM has a good synergy effect for metalloenzyme-producing strains, which is consistent with some previous studies (18). A previous study in China demonstrated that when avibactam is combined, the MIC_50_ and MIC_90_ of ATM against NDM-producing strains could be reduced by 99.9% and 99.8%, respectively.

Fosfomycin is infrequently used in clinical practice primarily for urinary tract infections since its first discovery (30). Therefore, it has maintained potent antibacterial activity against many multidrug-resistant pathogens including CRE. In addition, the drug has a low protein binding rate and a high tissue penetration rate (31), which give it the potential utility in combination antibiotic drug against MDR pathogens. FOS acts as a time-dependent inhibitor of the MurA enzyme, which catalyzes the first committed step of peptidoglycan synthesis (32). It has a synergy effect with CZA, probably because they act on different times of bacterial cell wall synthesis.

Compared with other studies, we could obtain similar conclusions. Firstly, drug combination is necessary for multi-drug resistant bacterial. For example, to preserve the effectiveness of CZA, its clinical use should be avoided in naturally resistant strains and in those carrying MBLs and certain class D β-carbapenemases (33). Combination therapy plays an important role in these clinical scenarios, as CZA showed a showed synergistic effect when combined with MEM or ATM. Those results are, in part, consistent with those of the previous studies (6, 33, 34). For KPC-producing strains, combination therapy also holds significant value. Some strains exhibit susceptibility to CZA but may present high MIC values (4 µg/mL or 8 µg/mL), approaching resistance levels. Utilizing CZA alone in treating such strains may lead to treatment failure. It’s been well-documented (35) that CZA in monotherapy of critically ill patients infected with carbapenem-producing *K. pneumoniae* has been associated with higher mortality than in combination therapy. In these scenarios (36), combination therapy can substantially influence the clinical outcome of the patient.

Our study has some advantages: we selected strains with multiple resistance mechanisms including MBL-producing and non-carbapenem-producing strains; in addition to KPC-producing strains, which is the only strains in other studies (37). This study has some limitations such as *in vivo* experiments are required to confirm our results. Further clinical studies are essential to evaluate the clinical impact of those combinations and establish the efficacy of those regimens in the treatment of infections due to CRKP isolates.

## SUPPLEMENTAL MATERIAL

Supplemental tables spectrum.00107-24-s0001.docx Tables S1 and S2.

ASM does not own the copyrights to Supplemental Material that may be linked to, or accessed through, an article. The authors have granted ASM a non-exclusive, world-wide license to publish the Supplemental Material files. Please contact the corresponding author directly for reuse.

## References

[1] CDC. 2019 Antibiotic Resistance Threats in the United States 2019. Available from: https://www. cdc.gov/drugresistance/pdf/threats-report/2019-ar-threats-report-508.pdf

[2] Rodríguez OL, Sousa A, Pérez-Rodríguez MT, Martínez-Lamas L, Suárez RL, Martínez CT, Pino CP, Vidal FV, Pérez-Landeiro A, Casal MC. 2021. Mortality-related factors in patients with OXA-48 carbapenemase-producing Klebsiella pneumoniae bacteremia. Medicine (Baltimore) 100:e24880. doi:10.1097/MD.0000000000024880 33832068 PMC8036053

[3] Liu E, Jia P, Li X, Zhou M, Kudinha T, Wu C, Xu Y, Yang Q. 2021. In vitro and in vivo effect of antimicrobial agent combinations against carbapenem-resistant Klebsiella pneumoniae with different resistance mechanisms in China. Infect Drug Resist 14:917–928. doi:10.2147/IDR.S292431 33707959 PMC7943327

[4] Yang Y, Yang Y, Chen G, Lin M, Chen Y, He R, Galvão KN, El-Gawad El-Sayed Ahmed MA, Roberts AP, Wu Y, Zhong L-L, Liang X, Qin M, Ding X, Deng W, Huang S, Li H-Y, Dai M, Chen D-Q, Zhang L, Liao K, Xia Y, Tian G-B. 2021. Molecular characterization of carbapenem-resistant and virulent plasmids in Klebsiella pneumoniae from patients with bloodstream infections in China. Emerg Microbes Infect 10:700–709. doi:10.1080/22221751.2021.1906163 33739229 PMC8023600

[5] Han R, Shi Q, Wu S, Yin D, Peng M, Dong D, Zheng Y, Guo Y, Zhang R, Hu F. 2020. Dissemination of carbapenemases (KPC, NDM, OXA-48, IMP, and VIM) among carbapenem-resistant enterobacteriaceae isolated from adult and children patients in China. Front Cell Infect Microbiol 10:314. doi:10.3389/fcimb.2020.00314 32719751 PMC7347961

[6] Mikhail S, Singh NB, Kebriaei R, Rice SA, Stamper KC, Castanheira M, Rybak MJ. 2019. Evaluation of the synergy of ceftazidime-avibactam in combination with meropenem, amikacin, aztreonam, colistin, or fosfomycin against well-characterized multidrug-resistant Klebsiella pneumoniae and Pseudomonas aeruginosa. Antimicrob Agents Chemother 63:e00779-19. doi:10.1128/AAC.00779-19 31182535 PMC6658738

[7] Shi Q, Yin D, Han R, Guo Y, Zheng Y, Wu S, Yang Y, Li S, Zhang R, Hu F. 2020. Emergence and recovery of ceftazidime-avibactam resistance in blaKPC-33-harboring Klebsiella pneumoniae sequence type 11 isolates in China. Clin Infect Dis 71:S436–S439. doi:10.1093/cid/ciaa1521 33367577

[8] Compain F, Arthur M. 2017. Impaired inhibition by avibactam and resistance to the ceftazidime-avibactam combination due to the D(179)Y substitution in the KPC-2 β-lactamase. Antimicrob Agents Chemother 61:e00451-17. doi:10.1128/AAC.00451-17 28461318 PMC5487616

[9] Shields RK, Chen L, Cheng S, Chavda KD, Press EG, Snyder A, Pandey R, Doi Y, Kreiswirth BN, Nguyen MH, Clancy CJ. 2017. Emergence of ceftazidime-avibactam resistance due to plasmid-borne bla(KPC-3) mutations during treatment of carbapenem-resistant Klebsiella pneumoniae infections. Antimicrob Agents Chemother 61:e02097-16. doi:10.1128/AAC.02097-16 28031201 PMC5328542

[10] Zhang Y, Zhao C, Wang Q, et al.. 2016. High prevalence of hypervirulent Klebsiella pneumoniae infection in China: geographic distribution clinical characteristics, and antimicrobial resistance. Antimicrob Agents Chemother 60:6115–6120. doi:10.1128/AAC.01127-16 27480857 PMC5038323

[11] Vamsi SK, Moorthy RS, Hemiliamma MN, Chandra Reddy RB, Chanderakant DJ, Sirikonda S. 2022. Phenotypic and genotypic detection of carbapenemase production among gram negative bacteria isolated from hospital acquired infections. Saudi Med J 43:236–243. doi:10.15537/smj.2022.43.3.20210809 35256490 PMC9280532

[12] van der Zwaluw K, de Haan A, Pluister GN, Bootsma HJ, de Neeling AJ, Schouls LM. 2015. The carbapenem inactivation method (CIM), a simple and low-cost alternative for the carba NP test to assess phenotypic carbapenemase activity in gram-negative rods. PLoS One 10:e0123690. doi:10.1371/journal.pone.0123690 25798828 PMC4370852

[13] Saravolatz LD, Pawlak J. 2022. In vitro activity of fosfomycin alone and in combination against Staphylococcus aureus with reduced susceptibility or resistance to methicillin, vancomycin, daptomycin or linezolid. J Antimicrob Chemother 78:238–241. doi:10.1093/jac/dkac380 36374572

[14] Bai Y, Liu B, Wang T, Cai Y, Liang B, Wang R, Liu Y, Wang J. 2015. In vitro activities of combinations of rifampin with other antimicrobials against multidrug-resistant Acinetobacter baumannii. Antimicrob Agents Chemother 59:1466–1471. doi:10.1128/AAC.04089-14 25534730 PMC4325818

[15] Milne KEN, Gould IM. 2010. Combination testing of multidrug-resistant cystic fibrosis isolates of Pseudomonas aeruginosa: use of a new parameter, the susceptible breakpoint index. J Antimicrob Chemother 65:82–90. doi:10.1093/jac/dkp384 19861334

[16] Shetty S, Shetty RM, et al.. 2021. Comparison of time-kill assay to evaluate the antimicrobial efficacy of garlic (Allium Sativum) and Guava (Psidium Guajava) extracts on periodontal pathogens. Contemp Clin Dent 12:389–395. doi:10.4103/ccd.ccd_731_20 35068838 PMC8740802

[17] Cebrero-Cangueiro T, Álvarez-Marín R, Labrador-Herrera G, Smani Y, Cordero-Matía E, Pachón J, Pachón-Ibáñez ME. 2018. In vitro activity of pentamidine alone and in combination with aminoglycosides, tigecycline, rifampicin, and doripenem against clinical strains of carbapenemase-producing and/or colistin-resistant enterobacteriaceae. Front Cell Infect Microbiol 8:363. doi:10.3389/fcimb.2018.00363 30406040 PMC6201057

[18] Yu W, Luo Q, Shen P, Chen Y, Xu H, Xiao Y, Qiu Y. 2021. New options for bloodstream infections caused by colistin- or ceftazidime/avibactam-resistant Klebsiella pneumoniae. Int J Antimicrob Agents 58:106458. doi:10.1016/j.ijantimicag.2021.106458 34706255

[19] Holt KE, Wertheim H, Zadoks RN, Baker S, Whitehouse CA, Dance D, Jenney A, Connor TR, Hsu LY, Severin J, et al.. 2015. Genomic analysis of diversity, population structure, virulence, and antimicrobial resistance in Klebsiella pneumoniae, an urgent threat to public health. Proc Natl Acad Sci U S A 112:E3574–81. doi:10.1073/pnas.1501049112 26100894 PMC4500264

[20] Xu L, Sun X, Ma X. 2017. Systematic review and meta-analysis of mortality of patients infected with carbapenem-resistant Klebsiella pneumoniae. Ann Clin Microbiol Antimicrob 16:18. doi:10.1186/s12941-017-0191-3 28356109 PMC5371217

[21] Schwaber MJ, Carmeli Y. 2007. Mortality and delay in effective therapy associated with extended-spectrum beta-lactamase production in enterobacteriaceae bacteraemia: a systematic review and meta-analysis. J Antimicrob Chemother 60:913–920. doi:10.1093/jac/dkm318 17848376

[22] Wang Y, Wang J, Wang R, Cai Y. 2020. Resistance to ceftazidime-avibactam and underlying mechanisms. J Glob Antimicrob Resist 22:18–27. doi:10.1016/j.jgar.2019.12.009 31863899

[23] Zhang P, Shi Q, Hu H, Hong B, Wu X, Du X, Akova M, Yu Y. 2020. Emergence of ceftazidime/avibactam resistance in carbapenem-resistant Klebsiella pneumoniae in China. Clin Microbiol Infect 26:124. doi:10.1016/j.cmi.2019.08.020 31494252

[24] Kazmierczak KM, Biedenbach DJ, Hackel M, Rabine S, de Jonge BLM, Bouchillon SK, Sahm DF, Bradford PA. 2016. Global dissemination of blaKPC into bacterial species beyond Klebsiella pneumoniae and in vitro susceptibility to ceftazidime-avibactam and aztreonam-avibactam. Antimicrob Agents Chemother 60:4490–4500. doi:10.1128/AAC.00107-16 27161636 PMC4958145

[25] Tumbarello M, Raffaelli F, Giannella M, Mantengoli E, Mularoni A, Venditti M, De Rosa FG, Sarmati L, Bassetti M, Brindicci G, et al.. 2021. Ceftazidime-avibactam use for Klebsiella pneumoniae carbapenemase-producing K. pneumoniae infections: a retrospective observational multicenter study. Clin Infect Dis 73:1664–1676. doi:10.1093/cid/ciab176 33618353

[26] Endimiani A, Patel G, Hujer KM, Swaminathan M, Perez F, Rice LB, Jacobs MR, Bonomo RA. 2010. In vitro activity of fosfomycin against blaKPC-containing Klebsiella pneumoniae isolates, including those nonsusceptible to tigecycline and/or colistin. Antimicrob Agents Chemother 54:526–529. doi:10.1128/AAC.01235-09 19901089 PMC2798518

[27] Cottagnoud P. 2002. Cellular and molecular aspects of drugs of the future: meropenem. Cell Mol Life Sci 59:1928–1933. doi:10.1007/pl00012515 12530523 PMC11337433

[28] Albiero J, Sy SKB, Mazucheli J, Caparroz-Assef SM, Costa BB, Alves JLB, Gales AC, Tognim MCB. 2016. Pharmacodynamic evaluation of the potential clinical utility of fosfomycin and meropenem in combination therapy against KPC-2-producing Klebsiella pneumoniae. Antimicrob Agents Chemother 60:4128–4139. doi:10.1128/AAC.03099-15 27139468 PMC4914646

[29] Drawz SM, Papp-Wallace KM, Bonomo RA. 2014. New β-lactamase inhibitors: a therapeutic renaissance in an MDR world. Antimicrob Agents Chemother 58:1835–1846. doi:10.1128/AAC.00826-13 24379206 PMC4023773

[30] Avery LM, Sutherland CA, Nicolau DP. 2019. In vitro investigation of synergy among fosfomycin and parenteral antimicrobials against carbapenemase-producing enterobacteriaceae. Diagn Microbiol Infect Dis 95:216–220. doi:10.1016/j.diagmicrobio.2019.05.014 31213392

[31] Grabein B, Graninger W, Rodríguez Baño J, Dinh A, Liesenfeld DB. 2017. Intravenous fosfomycin-back to the future. systematic review and meta-analysis of the clinical literature. Clin Microbiol Infect 23:363–372. doi:10.1016/j.cmi.2016.12.005 27956267

[32] Silver LL. 2017. Fosfomycin: mechanism and resistance. Cold Spring Harb Perspect Med 7:a025262. doi:10.1101/cshperspect.a025262 28062557 PMC5287057

[33] Montero MM, Domene Ochoa S, López-Causapé C, Luque S, Sorlí L, Campillo N, López Montesinos I, Padilla E, Prim N, Angulo-Brunet A, Grau S, Oliver A, Horcajada JP. 2021. Time-kill evaluation of antibiotic combinations containing ceftazidime-avibactam against extensively drug-resistant Pseudomonas aeruginosa and their potential role against ceftazidime-avibactam-resistant isolates. Microbiol Spectr 9:e0058521. doi:10.1128/spectrum.00585-21 34319141 PMC8552783

[34] Mantzana P, Protonotariou E, Kassomenaki A, Meletis G, Tychala A, Keskilidou E, Arhonti M, Katsanou C, Daviti A, Vasilaki O, Kagkalou G, Skoura L. 2023. In vitro synergistic activity of antimicrobial combinations against carbapenem- and colistin-resistant Acinetobacter baumannii and Klebsiella pneumoniae. Antibiotics (Basel) 12:93. doi:10.3390/antibiotics12010093 36671295 PMC9855173

[35] Davido B, Crémieux A-C, Vaugier I, Gatin L, Noussair L, Massias L, Laurent F, Saleh-Mghir A. 2023. Efficacy of ceftazidime-avibactam in various combinations for the treatment of experimental osteomyelitis due to Klebsiella pneumoniae carbapenemase (KPC)-producing Klebsiella pneumoniae. Int J Antimicrob Agents 61:106702. doi:10.1016/j.ijantimicag.2022.106702 36476965

[36] Zheng G, Zhang J, Wang B, Cai J, Wang L, Hou K, Zhang Y, Zhang L, Yang Z, He J, Bian X. 2021. Ceftazidime-avibactam in combination with in vitro non-susceptible antimicrobials versus ceftazidime-avibactam in monotherapy in critically ill patients with carbapenem-resistant Klebsiella pneumoniae infection: a retrospective cohort study. Infect Dis Ther 10:1699–1713. doi:10.1007/s40121-021-00479-7 34241831 PMC8322179

[37] Bianco G, Boattini M, Comini S, Iannaccone M, Bondi A, Cavallo R, Costa C. 2022. In vitro activity of cefiderocol against ceftazidime-avibactam susceptible and resistant KPC-producing enterobacterales: cross-resistance and synergistic effects. Eur J Clin Microbiol Infect Dis 41:63–70. doi:10.1007/s10096-021-04341-z 34462816

