# Supplementary material for: Effect of ceftazidime-avibactam combined with different antimicrobials against carbapenem-resistant Klebsiella pneumoniae
Source: Microbiol Spectr. 2024 May 7;12(6):e00107-24. doi: 10.1128/spectrum.00107-24 (PMC11237391; doi:10.1128/spectrum.00107-24)
Supplement: Supplemental tables — Tables S1 and S2. [file spectrum.00107-24-s0001.docx]

**Supplement Table1. Informations of 59 isolates in this study**

| Strain No | Carbapenemase | ST type | Specimen | Strain No | Carbapenemase | ST type | Specimen |
| --- | --- | --- | --- | --- | --- | --- | --- |
| P1 | KPC-3 | - | sputum | Y081 | - | ST-4392 | drainage |
| P2 | KPC-4 | - | sputum | Y083 | - | ST-4933 | blood |
| D07 | KPC-2 | ST-69 | BALF | Y105 | - | ST-4928 | blood |
| D16 | KPC-2 | ST-14 | BALF | C72 | NDM-1 | ST4927 | sputum |
| D52 | KPC-2 | ST-65 | sputum | C74 | NDM-1 | ST20 | drainage |
| D56 | KPC-2 | ST-147 | bile | C280 | NDM-1 | ST1711 | abscess |
| H057 | KPC-2 | ST-37 | abscess | C281 | NDM-1 | ST1711 | abscess |
| H104 | KPC-2 | ST-15 | urine | C295 | NDM-1 | ST11 | drainage |
| L118 | KPC-2 | ST-1797 | drainage | L4 | NDM-1 | ST25 | drainage |
| L123 | KPC-2 | ST-11 | blood | L014 | NDM-1 | ST11 | drainage |
| L143 | KPC-2 | ST-494 | blood | L013 | NDM-1 | ST11 | bile |
| L144 | KPC-2 | ST-11 | urine | L03 | NDM-1 | ST25 | drainage |
| L145 | KPC-2 | ST-11 | drainage | C12 | NDM-1 | ST4927 | BALF |
| L40 | KPC-2 | ST-13 | sputum | C87 | NDM-1 | ST20 | drainage |
| L48 | KPC-2 | ST-655 | sputum | L1 | NDM-1 | ST1 | drainage |
| L52 | KPC-2 | ST-22 | urine | CD2 | NDM-5 | - | blood |
| L60 | KPC-2 | ST-412 | sputum | CD11 | NDM-5 | - | blood |
| L70 | KPC-2 | ST-48 | blood | L107 | IMP-4 | ST454 | drainage |
| L80 | KPC-2 | ST-11 | bile | H030 | IMP-4 | ST48 | urine |
| L90 | KPC-2 | ST-656 | urine | L33 | IMP-4 | ST17 | blood |
| R37 | KPC-2 | ST-1 | drainage | L034 | IMP-4 | ST17 | blood |
| Y070 | KPC-2 | ST-23 | blood | L112 | IMP-4 | ST54 | drainage |
| H004 | - | ST-4928 | abscess | Y077 | IMP-4 | ST416 | drainage |
| H029 | - | ST-4928 | urine | Y076 | IMP-4 | ST416 | blood |
| L093 | - | ST-4929 | bile | H027 | IMP-26 | ST1697 | abscess |
| Y008 | - | ST-4930 | drainage | Y049 | IMP-8 | ST17 | urine |
| Y061 | - | ST-4930 | csf | Y047 | IMP-8 | ST17 | urine |
| Y067 | - | ST-4926 | blood | L94 | IMP-8 | ST11 | drainage |
| Y068 | - | ST-4930 | drainage | H031 | IMP-8 | ST48 | urine |
| Y075 | - | ST-4931 | blood |  |  |  |  |

**Abbreviations：BALF, bronchoalveolar lavage fluid; csf, cerebrospinal fluid.**

**Supplement Table2. Detailed results of Antimicrobial susceptibility test and Checkerboard Test**

| Strain No | Carbapenema-se | MIC(mg/L) | | | | | | | CZA+AK | CZA+COL | CZA+ERA | CZA+FOS | CZA+MEM | CZA+ATM |
| --- | --- | --- | --- | --- | --- | --- | --- | --- | --- | --- | --- | --- | --- | --- |
|  |  | CZA | AK | COL | ERA | FOS | MEM | ATM | FICI | FICI | FICI | FICI | FICI | FICI |
| P1 | KPC-3 | 2 | 128 | 0.25 | 1 | 512 | 128 | - | 0.250 | 1.000 | 0.312 | 0.281 | 0.019 | - |
| P2 | KPC-4 | 0.125 | 1 | 16 | 1 | 512 | 0.5 | - | 0.375 | 0.625 | 0.312 | 0.253 | 0.750 | - |
| D07 | KPC-2 | 0.5 | 16 | 0.25 | 0.5 | 4 | 8 | - | 0.625 | 0.750 | 0.625 | 1.000 | 0.063 | - |
| D16 | KPC-2 | 0.5 | 4 | 0.25 | 2 | 64 | 32 | - | 1.250 | 3.000 | 1.250 | 0.500 | 0.040 | - |
| D52 | KPC-2 | 0.5 | 128 | 0.25 | 0.5 | 256 | 8 | - | 1.5 | 2.500 | 1.125 | 1.250 | 0.063 | - |
| D56 | KPC-2 | 0.25 | 1 | 0.25 | 0.5 | 1024 | 16 | - | 0.750 | 1.500 | 1.125 | 1.002 | 0.080 | - |
| H057 | KPC-2 | 0.5 | 128 | 0.25 | 0.125 | 128 | 32 | - | 1.010 | 3.000 | 3.000 | 0.500 | 0.040 | - |
| H104 | KPC-2 | 0.5 | 0.5 | 0.25 | 1 | 4 | 16 | - | 0.750 | 1.250 | 1.250 | 0.750 | 0.048 | - |
| L118 | KPC-2 | 0.5 | 128 | 0.25 | 0.25 | 512 | 32 | - | 1.250 | 0.750 | 0.500 | 0.750 | 0.040 | - |
| L123 | KPC-2 | 1 | 128 | 0.125 | 0.25 | 512 | 128 | - | 1.010 | 1.500 | 1.000 | 0.750 | 0.018 | - |
| L143 | KPC-2 | 1 | 0.5 | 0.25 | 0.5 | 1024 | 8 | - | 1.250 | 1.250 | 1.125 | 1.250 | 0.047 | - |
| L144 | KPC-2 | 2 | 0.5 | 0.25 | 0.25 | 1024 | 128 | - | 1.006 | 0.250 | 1.250 | 0.330 | 0.010 | - |
| L145 | KPC-2 | 2 | 128 | 0.25 | 0.5 | 1024 | 128 | - | 1.003 | 1.125 | 1.250 | 0.500 | 0.010 | - |
| L40 | KPC-2 | 0.5 | 128 | 0.125 | 0.125 | 16 | 128 | - | 0.750 | 2.500 | 0.750 | 0.625 | 0.034 | - |
| L48 | KPC-2 | 1 | 128 | 0.25 | 0.25 | 64 | 32 | - | 0.625 | 0.625 | 1.500 | 1.000 | 0.024 | - |
| L52 | KPC-2 | 1 | 0.5 | 0.25 | 0.25 | 512 | 8 | - | 1.500 | 1.125 | 1.250 | 0.500 | 0.047 | - |
| L60 | KPC-2 | 0.5 | 1 | 0.25 | 0.25 | 512 | 16 | - | 0.500 | 1.500 | 0.625 | 0.375 | 0.048 | - |
| L70 | KPC-2 | 0.031 | 128 | 0.25 | 0.5 | 8 | 8 | - | 0.254 | 0.750 | 0.750 | 0.750 | 0.547 | - |
| L80 | KPC-2 | 4 | 128 | 0.25 | 0.25 | 1024 | 128 | - | 1.000 | 1.250 | 0.625 | 1.03 | 0.006 | - |
| L90 | KPC-2 | 1 | 1 | 0.25 | 1 | 256 | 32 | - | 0.625 | 2.5 | 0.564 | 1 | 0.024 | - |
| R37 | KPC-2 | 0.25 | 128 | 0.25 | 0.25 | 1024 | 32 | - | 1.03 | 2.5 | 1 | 0.5 | 0.080 | - |
| Y070 | KPC-2 | 2 | 128 | 0.25 | 0.25 | 1024 | 128 | - | 1.03 | 1.5 | 0.564 | 0.5 | 0.012 | - |

**Continuation table**

| Strain No | Carbapenema-se | MIC(mg/L) | | | | | | | CZA+AK | CZA+COL | CZA+ERA | CZA+FOS | CZA+MEM | CZA+ATM |
| --- | --- | --- | --- | --- | --- | --- | --- | --- | --- | --- | --- | --- | --- | --- |
|  |  | CZA | AK | COL | ERA | FOS | MEM | ATM | FICI | FICI | FICI | FICI | FICI | FICI |
| H004 | - | 1 | 128 | 2 | 0.5 | 256 | 8 | - | 2.002 | 1.5 | 1.125 | 0.625 | 0.047 | - |
| H029 | - | 0.25 | 128 | 0.125 | 2 | 32 | 4 | - | 1.002 | 1.25 | 1.5 | 1 | 0.127 | - |
| L093 | - | 4 | 0.5 | 0.25 | 4 | 256 | 32 | - | 1.06 | 1.25 | 0.625 | 0.5 | 0.020 | - |
| Y008 | - | 0.25 | 4 | 0.125 | 0.5 | 32 | 8 | - | 1.000 | 1.500 | 0.625 | 0.56 | 0.095 | - |
| Y061 | - | 1 | 128 | 0.25 | 4 | 512 | 32 | - | 1.002 | 1.125 | 0.75 | 0.375 | 0.024 | - |
| Y067 | - | 1 | 128 | 0.25 | 0.25 | 128 | 128 | - | 1.002 | 1.25 | 0.625 | 0.56 | 0.010 | - |
| Y068 | - | 0.5 | 128 | 0.5 | 1 | 1024 | 8 | - | 1.002 | 1.25 | 0.314 | 1.25 | 0.063 | - |
| Y075 | - | 0.5 | 4 | 0.25 | 1 | 1024 | 4 | - | 0.75 | 2 | 1 | 0.502 | 0.095 | - |
| Y081 | - | 0.5 | 2 | 0.25 | 1 | 1024 | 2 | - | 1 | 1.125 | 1 | 1.002 | 0.157 | - |
| Y083 | - | 4 | 128 | 0.25 | 0.25 | 128 | 16 | - | 0.516 | 0.75 | 0.5 | 0.375 | 0.020 | - |
| Y105 | - | 4 | 128 | 1 | 1 | 64 | 16 | - | 1.002 | 1.015 | 0.5625 | 0.625 | 0.020 | - |
| C72 | NDM-1 | 64 | 1 | 0.25 | 0.25 | 256 | 4 | 64 | 0.563 | 1.002 | 0.750 | 0.625 | 0.188 | 0.006 |
| C74 | NDM-1 | 64 | 64 | 0.25 | 0.5 | 512 | 64 | 64 | 0.033 | 1.002 | 1.002 | 1.500 | 0.500 | 0.006 |
| C280 | NDM-1 | 64 | 2 | 1 | 0.5 | 128 | 64 | 64 | 1.002 | 0.504 | 1.002 | 0.252 | 0.254 | 0.127 |
| C281 | NDM-1 | 2 | 64 | 0.25 | 2 | 512 | 64 | 64 | 0.750 | 0.750 | 0.531 | 0.563 | 0.078 | 0.094 |
| C87 | NDM-1 | 64 | 2 | 0.5 | 0.5 | 512 | 64 | 64 | 0.750 | 0.750 | 1.002 | 0.064 | 0.313 | 0.006 |
| C295 | NDM-1 | 2 | 64 | 0.25 | 4 | 512 | 64 | 64 | 1.004 | 1.063 | 0.750 | 0.375 | 0.066 | 0.078 |
| L4 | NDM-1 | 64 | 2 | 0.25 | 0.25 | 32 | 64 | 64 | 0.502 | 1.002 | 1.002 | 0.502 | 1.500 | 0.006 |
| L014 | NDM-1 | 64 | 4 | 0.25 | 0.5 | 256 | 64 | 64 | 0.502 | 1.002 | 1.002 | 0.252 | 0.188 | 0.018 |
| L013 | NDM-1 | 64 | 8 | 0.25 | 0.5 | 512 | 64 | 64 | 0.252 | 1.002 | 1.002 | 0.127 | 0.129 | 0.006 |

**Continuation table**

| Strain No | Carbapenema-se | MIC(mg/L) | | | | | | | CZA+AK | CZA+COL | CZA+ERA | CZA+FOS | CZA+MEM | CZA+ATM |
| --- | --- | --- | --- | --- | --- | --- | --- | --- | --- | --- | --- | --- | --- | --- |
|  |  | CZA | AK | COL | ERA | FOS | MEM | ATM | FICI | FICI | FICI | FICI | FICI | FICI |
| L03 | NDM-1 | 64 | 2 | 0.25 | 0.25 | 256 | 64 | 64 | 0.502 | 1.002 | 1.002 | 0.127 | 1.500 | 0.006 |
| C12 | NDM-1 | 64 | 1 | 0.25 | 1 | 512 | 64 | 64 | 1.002 | 1.002 | 0.502 | 0.127 | 0.156 | 0.010 |
| L1 | NDM-1 | 64 | 64 | 1 | 1 | 512 | 64 | 64 | 0.033 | 0.502 | 1.002 | 0.127 | 0.156 | 0.033 |
| CD2 | NDM-5 | 64 | 2 | 0.5 | 1 | 512 | 64 | 64 | 0.502 | 0.502 | 1.002 | 0.127 | 1.500 | 0.006 |
| CD11 | NDM-5 | 64 | 64 | 0.5 | 2 | 512 | 64 | 64 | 1.500 | 1.002 | 2.002 | 0.502 | 1.500 | 0.010 |
| L107 | IMP-4 | 64 | 1 | 2 | 0.5 | 512 | 8 | 16 | 1.002 | 0.141 | 0.187 | 0.127 | 1.002 | 0.133 |
| H030 | IMP-4 | 64 | 2 | 0.25 | 2 | 256 | 32 | 64 | 0.252 | 1.002 | 1.002 | 0.127 | 0.023 | 0.006 |
| L33 | IMP-4 | 64 | 1 | 0.25 | 0.5 | 512 | 8 | 1 | 0.625 | 0.498 | 0.374 | 0.127 | 0.156 | 0.252 |
| L034 | IMP-4 | 64 | 1 | 0.25 | 0.5 | 512 | 16 | 2 | 1.004 | 0.498 | 0.374 | 0.254 | 0.750 | 0.129 |
| L112 | IMP-4 | 64 | 1 | 0.5 | 0.25 | 256 | 2 | 64 | 1.004 | 0.500 | 1.004 | 0.375 | 0.500 | 0.008 |
| Y077 | IMP-4 | 64 | 1 | 0.25 | 0.5 | 512 | 8 | 16 | 0.750 | 1.004 | 0.563 | 0.035 | 0.500 | 0.035 |
| Y076 | IMP-4 | 64 | 4 | 0.5 | 0.5 | 512 | 4 | 16 | 0.375 | 0.504 | 0.504 | 2.000 | 0.188 | 0.035 |
| H027 | IMP-26 | 64 | 2 | 0.5 | 8 | 512 | 32 | 64 | 0.252 | 1.002 | 1.002 | 0.252 | 0.125 | 0.006 |
| Y049 | IMP-8 | 64 | 64 | 0.25 | 0.25 | 256 | 2 | 64 | 2.000 | 0.498 | 1.004 | 0.129 | 0.375 | 0.008 |
| Y047 | IMP-8 | 64 | 64 | 2 | 1 | 512 | 4 | 64 | 2.000 | 0.254 | 0.375 | 0.066 | 0.125 | 0.008 |
| L94 | IMP-8 | 64 | 64 | 2 | 1 | 128 | 8 | 16 | 2.000 | 0.254 | 1.004 | 1.000 | 0.500 | 0.020 |
| H031 | IMP-8 | 64 | 0.5 | 0.25 | 4 | 512 | 32 | 32 | 0.504 | 1.004 | 1.004 | 0.504 | 0.129 | 0.012 |
